# Performance of central venous catheterization by medical students: a retrospective study of students’ logbooks

**DOI:** 10.1186/1472-6920-14-168

**Published:** 2014-08-13

**Authors:** Anne Chao, Chia-Hsin Lai, Kuang-Cheng Chan, Chi-Chuan Yeh, Hui-Ming Yeh, Shou-Zen Fan, Wei-Zen Sun

**Affiliations:** 1 Department of Anesthesiology, National Taiwan University Hospital, 7, Chung-Shan South Road, Taipei 100, Taiwan; 2 Department of Anesthesiology, Taiwan Adventist Hospital, 424, Section 2, Bade Road, Songshan District, Taipei 105, Taiwan; 3 Department of Medical Education, National Taiwan University Hospital, 7, Chung-Shan South Road, Taipei 100, Taiwan

**Keywords:** Central venous catheterization, Logbook, Medical student

## Abstract

**Background:**

Medical students often learn the skills necessary to perform a central venous catheterization in the operating room after simulator training. We examined the performance of central venous catheterization by medical students from the logbooks during their rotation in department of anesthesiology.

**Methods:**

From the logbooks of medical students rotating in our department between January 2011 and June 2012, we obtained the kind and the number of central venous catheterization students had done, the results of the procedures whether they were success or failed, the reasons of the failures, complications, and the student self-reported confidence and satisfaction of their performance.

**Results:**

There were 93 medical students performed 875 central venous catheterizations with landmark guidance on patients in the operating theater, and the mean number of catheterizations performed per student was 9.4 ± 2.0, with a success rate of 67.3%. Adjusted for age, sex, body mass index, surgical category, ASA score and insertion site, the odds of successful catherization improved with cumulative practice (odds ratio 1.10 per additional central venous catheterization performed; 95% confidence interval 1.05–1.15). The major challenge students encountered during the procedure was the difficulty of finding the central veins, which led to 185 catheterizations failed. The complication rate of central venous catheterization by the students was 7.8%, while the most common complication was puncture of artery. The satisfaction and confidence of students regarding their performance increased with each additional procedure and decreased significantly if failure or complications had occurred.

**Conclusion:**

A student logbook is a useful tool for recording the actual procedural performance of students. From the logbooks, we could see the students’ performance, challenges, satisfaction and confidence of central venous catheterization were improved through cumulative clinical practice of the procedure.

## Background

Central venous catheterization (CVC) is a basic clinical skill of doctors working in the departments of internal medicine, surgery, critical care, pediatrics, anesthesiology, and emergency. Prior studies had suggested that formal instruction in emergency skills is important, since it resulted in improved doctors’ procedural competence and reduced the rate of fatal errors [1,2]. Unfortunately, many residents reported a lack of confidence in performing the invasive procedures to their patients [3]. Accordingly, CVC is taught to medical students during their rotation in our anesthesiology department for training these future residents to perform the procedure competently in their career.

In the beginning of their course, medical students received CVC simulation training. Similar to other studies [4,5], we use a checklist as the competence assessment tool for CVC. After passing the assessment, then the simulator-trained medical students performed CVCs on patients in the operating theater. Teaching this invasive skill to medical students has been an important task in our anesthesiology department. We requested all the students to record every CVC they performed in their logbooks which were given to them at the beginning of their course in anesthesiology.

The main research question of this study: What was the performance of CVC by the simulator-trained medical students in the operating theatre? At the same time, we would like to know about medical students’ self-evaluated satisfaction and confidence regarding their performance.

## Methods

### Participants

All the data used in this study were obtained from the logbooks of all the 93 last-year medical students who underwent a three- to four-week rotation in our department between January 2011 and June 2012.

### Training course

On the first day, all students received a lecture including the introduction to the program, demonstration of central venous catheter kits, and watching an anesthesiologist perform the procedure, explaining CVC indications and potential complications. The supervisor demonstrated CVC with anatomic landmark-guided techniques [6]. After the session, students started individual simulation practice by using manikins under the guidance of a supervisor in the clinical skills center of the hospital. The supervisor gave students a procedural checklist to remind them to follow the steps of CVC. Besides learning the procedural skills, students also learned the proper pre-CVC preparation: wearing a mask and a cap, hand washing, putting on a gown and gloves; the infection control: cleaning the skin with chlorhexidine, and using full-barrier precautions during the insertion of central venous catheters; the team-work: adjusting proper simulator’s position, opening the CVC kit, passing required items needed and the post-procedural care: suturing and covering the catheter, putting away the used CVC kit, and placing sharp and pointed objects into a tin. After two days of repetitive practice on manikins, a supervisor evaluated each student by watching them from the beginning to the end of the procedure with the checklist. If they did poorly, they had to practice on the manikins until they passed the assessment.

In the operating room, we encouraged each medical student to perform 10 catheterizations. Students were not allowed to perform the procedure on patients with American Society of Anesthesiologists (ASA) physical status greater than III. Before starting the procedure, students first reviewed the medical charts of the patients to look at weight, height, type of surgery to be performed, ASA physical status, and laboratory data, and then they prepared the patient and started the procedure. After completing the procedure, a supervisor gave feedbacks to the student. If the student failed the procedure, the supervisor discussed the reasons of failure with the student and gave advice to improve the skill.

### Data collection

The data of the performance of CVC by medical students, such as the success rate, reasons of failure, complication rate, type of complication, and basic data of the patients they performed upon could be found in the logbooks of medical students. Other feedbacks from students, self-reported satisfaction and confidence regarding their procedural performance were also available.

Students recorded the demographics of the patients and the procedures whether they were successful or failed on the logbook. The procedure was defined as a success if it was completed solely by the student with or without verbal instructions from the supervisor, whereas it was considered failed if the catheterization was taken over by the supervisor or part of the procedure was performed by the supervisor. If they failed the procedure, they needed to record the reason of failure. Students had to examine and record every patient to see if any complications had occurred at the end of the procedure. Students’ self-evaluated satisfaction with their performance was also recorded [from 1 (least satisfied) to 4 (highly satisfied)]. They were asked to grade their confidence about performing future CVCs [from 1 (not confident) to 4 (highly confident)].

### Data analysis

The baseline characteristics: patient demographics, surgical categories, CVC insertion sites, complications, failure reasons, performance satisfaction and confidence in next CVC were reported as numbers and percentages or means ± standard deviations in Table 1. We would like to know the variables that might affect the success of CVC, students’ satisfaction and confidence; therefore univariate analysis was performed using a simple generalised linear model for categorical and continuous variables. The results were expressed as odds ratios with their 95% confidence intervals. The significant variables were then included in the multivariate analysis using a multiple generalised linear model. The method of generalised estimating equations was used to correlate the response data. Two-tailed *p*-values < .05 were considered statistically significant. Statistical analyses were performed with R software for Windows, version 2.12.0 (copyright 2010, The R Foundation for Statistical Computing).

**Table 1:** Differences between successful and failed CVC performance of medical students

**Variable**	**Failure**		**Success**	
	**N = 286**	**Percentage**	**N = 589**	**Percentage**
Sex				
Female	125	0.44	249	0.43
Male	160	0.56	336	0.57
ASA score				
I	19	0.07	28	0.05
II	118	0.42	256	0.45
III	145	0.51	289	0.50
Specialty				
General surgery	154	0.53	349	0.59
Neurosurgery	55	0.19	82	0.14
Chest surgery	33	0.12	63	0.11
Urology surgery	17	0.06	44	0.07
Ear-nose-throat surgery	18	0.06	21	0.04
Cardiovascular surgery	5	0.02	17	0.03
Gynaecology surgery	1	0.01	6	0.01
Plastic surgery	2	0.01	2	0.01
Orthopaedic surgery	0	0	3	0.01
Site of central vein				
Right internal jugular	187	0.65	448	0.70
Right femoral	44	0.15	67	0.11
Left femoral	39	0.14	50	0.09
Left internal jugular	16	0.06	23	0.04
Complications				
Arterial puncture	33	0.72	13	0.59
Hematoma	13	0.28	6	0.27
Pneumothorax	0	0	3	0.14
Performance satisfaction				
Disappointed	36	0.13	8	0.01
Not satisfied	170	0.61	166	0.3
Satisfied	49	0.18	243	0.43
Highly satisfied	19	0.07	145	0.26
Confidence				
Not confident at all	36	0.13	18	0.03
Slightly confident	141	0.51	197	0.35
Confident	95	0.35	323	0.57
Highly confident	3	0.01	24	0.04
Failure reason				
Failed to find vein	185	0.65		
Failed to pass guide wire	95	0.33		
Miscellaneous	6	0.02		

CVC: Central venous catheterization.

### Ethical approval

The Institutional Review Board of the National Taiwan University Hospital had approved this study (Case number: 201212002RINC). Since this was a retrospective study, the board waived the informed consents from medical students and patients included in this research.

## Results

In total, 93 students performed 875 CVCs during the 18-month study period, and the mean number of CVCs performed per student was 9.4 ± 2.0 (3 CVCs to 15 CVCs). The overall success rate of the CVCs carried out by medical students was 67.3% (589/875 patients). The highest success rate of the catheterization of central vein was in the right internal jugular vein (70.6%), followed by the right femoral vein (60.4%), the left internal jugular vein (59%), and the left femoral vein (56.2%). We listed the differences between the successful CVCs and the failed CVCs in terms of patient characteristics, surgery categories, insertion sites, complications, students’ satisfaction and students’ confidence in Table 1.

From Table 1, the overall complication rate of CVC performed by medical students was 7.8% (68/875). The most frequent complication was the puncture of the artery, which occurred in 46 patients. It comprised of 68% of the complications and 5.2% of all the CVCs performed by medical students. The next common complication was the presence of a significant hematoma, which happened in 19 patients, 28% of the complications and 2.2% of all CVCs performed. Pneumothorax occurred in 3 patients, 4% of the complications and 0.3% of all the CVCs performed.

The most common reason for catheterization failure was the inability to find the central vein, which occurred in 185 cases (64.7% of the failed CVCs). The next common reason was the inability to pass the guidewire through the puncture needle into the vein in 95 cases (33.2% of failed CVCs). Six cases failed because of miscellaneous reasons, including a thrombosed vein (detected with ultrasound later), supervisor took over the procedure because of student taking too much time, and the contamination of gloves and contents of the central venous catheter kits.

**Figure 1:**
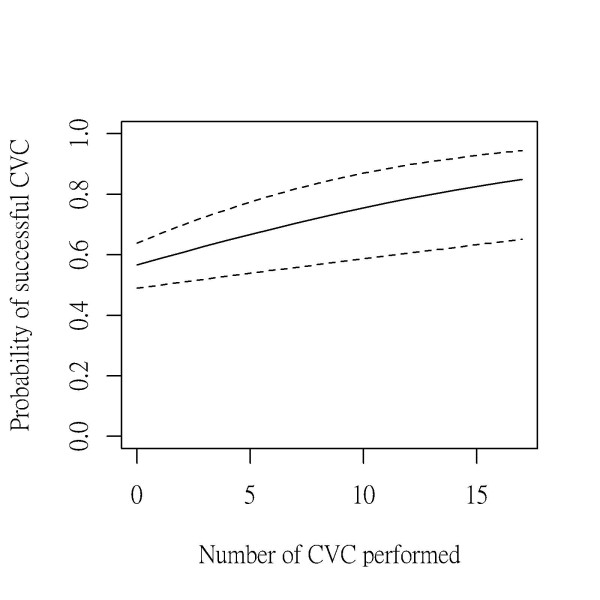
**Learning curve of the central venous catheterization by medical students.** The probability of successful catheterization (solid line) and the 95% confidence interval (broken lines) are also displayed.

From the simple univariate analysis, the number of CVC practiced and the access site of catheterization were factors likely to affect the success of CVC. The factors remained significant after multivariate analysis. Table 2 showed that with every additional CVC performed by students, the subsequent catheterization was 1.10-fold more likely to be successful. The success rate decreased 0.51-, 0.58-, and 0.55-fold, respectively, if the left femoral, right femoral, and left internal jugular vein were chosen as the access site compared with the right internal jugular vein approach. Figure 1 showed the improvement of procedural skills of medical students over time.

**Table 2:** Association between successful CVC and patient characteristics

**Variable**	**Univariate analysis**		**Multivariate analysis**	
	**Odds ratio**	***p*-value**	**Odds ratio**	***p*-value**
Number of CVCs	1.09 (1.04 ~ 1.14)	<0.001^*^	1.10 (1.05 ~ 1.15)	<0.001^*^
Age	1.01 (1.00 ~ 1.01)	0.234		
Sex				
Female	1.09 (0.84 ~ 1.42)	0.526		
Male	1.00			
Height	1.01 (0.99 ~ 1.02)	0.468		
Weight	1.00 (0.99 ~ 1.01)	0.826		
Body mass index	1.00 (0.97 ~ 1.03)	0.960		
ASA score	1.02 (0.81 ~ 1.27)	0.880		
Site of central vein				
Left femoral	0.51 (0.31 ~ 0.83)	0.006^*^	0.51 (0.31 ~ 0.83)	0.006^*^
Right femoral	0.58 (0.38 `0.89)	0.012^*^	0.58 (0.38 ~ 0.89)	0.012^*^
Left internal jugular	0.55 (0.27 ~ 1.12)	0.100	0.55 (0.27 ~ 1.12)	0.100
Right internal jugular	1.00			

^*^p < 0.05.

CVC: Central venous catheterization.

We also found that the percentage of students’ self-evaluated satisfaction regarding their performance was higher in successful CVCs than the failed CVCs (69% vs 25%, Table 1). Factors affecting their satisfaction scores were number of CVCs performed, failure of catheterization and presence of complications. Table 3 showed that every additional CVC done, satisfaction sores increased 0.06 points, while presence of failure and complications, the scores decreased by 0.71 and 0.69 points respectively (Table 3). Similarly, Table 4 showed that the scores of students’ confidence for future CVC increased with the number of catheterizations performed (0.05 points with every additional CVC done) and decreased with unsuccessful catheterizations (0.36 points) and the occurrence of complications (0.51 points).

**Table 3:** Medical student satisfaction of CVC performance

**Variable**	**Coefficient**	***p*-value**
Number of CVCs performed	0.065 (0.04 ~ 0.08)	<0.001
Failure		
No	0	
Yes	-0.71 (-0.80 ~ -0.57)	<0.001
Complications		
No	0	
Yes	-0.69 (-0.86 ~ -0.52)	<0.001

CVC: Central venous catheterization.

**Table 4:** Medical student confidence for next CVC

**Variable**	**Coefficient**	***p*-value**
Number of CVCs performed	0.05 (0.04 ~ 0.07)	<0.001
Failure		
No	0	
Yes	-0.36 (-0.50 ~ -0.23)	<0.001
Complications		
No	0	
Yes	-0.51 (-0.73 ~ -0.30)	<0.001

CVC: Central venous catheterization.

## Discussion

Perform CVC successfully in a safe and sterile way for patients is our goal to teach medical students. However, either our teachers or students do not consider our success rate of 67.3% satisfactory, thus we should investigate the performance of students and make improvements. There are two ways to solve this problem. First, increase actual practice in clinical setting. According to the extrapolation of our learning curve shown in Figure 1, in order to increase the success rate of the skill to 80% or higher, students probably would have to perform more than 15 CVCs. This would be a difficult task to achieve. Medical students face limited opportunities to perform CVCs on patients. In the operating theater, patients considered at risk of developing complications are not eligible for students to perform the procedure upon, such as small children, patients with ASA physical status IVE, patients with coagulopathy and those with poor anatomic landmarks. Many patients receive peripheral large bore intravenous lines instead of central catheters for temporary use. As such, the number of CVC opportunities suitable for medical students is limited.

Increase clinical experience in the operating room is difficult, so the alternate solution is to spend more time on simulation training. Despite the major drawback of practicing on manikins is the lack of variations that is different from real patients, students can still improve their dexterity through simulation training. Previous studies showed that simulation-based training improved the central venous catheter insertion and advanced cardiac life support skills of internal medicine residents and medical students [4,7]. Some students felt awkward using one hand for advancing the needle into the vein. On manikins, they can learn how to hold a puncture needle single-handed to advance the needle into a central vein. Once they have learned it, when they perform CVC on patients, one of the hands advancing needle, another hand either palpating the artery or holding an ultrasound probe to guide the needle into the central vein. This may increase the success rate. After students have placed the puncture needle in the vein, they have to learn how to keep the needle in the vessel steadily. Students should not advance the guidewire with force, since this could easily cause the guidewire kinked. Students should learn how to cut and dilate skin and soft tissues properly, and then they can place the catheter over the guidewire smoothly. We need to focus on the common reasons why previous students failed the procedure and let future students deliberately practice these steps in the simulation center. CVC skills may be acquired more rapidly if students learn sequentially in smaller steps and then combine them into one, seamless performance.

In this study, the complications rate was 7.8%, which was similar to the published rates of iatrogenic mechanical complications associated with the catheterisation of the internal jugular vein (6.3% to 11.8%) and femoral vein (12.8% to 19.4%) [8-11]. Several studies demonstrated that ultrasound-guided CVCs had a significantly higher success rate and lower complications rate than procedures where ultrasound is not used [12-15]. We will incorporate ultrasound-guided CVC teaching into the medical student training.

As this is a retrospective study, it has certain limitations. Firstly, the logbook is a tool for recording student actual clinical performance. Because students recorded the data themselves, it was difficult for students to time the procedure, to count the number of sticks they had done. Therefore, some detailed data were not available in this study. Secondly, the attitude of students was not included. From the logbooks of two medical students, they performed only three CVCs in operating theater. We do not know the reason why. It might be they had low motivation to perform the procedure, they did not have the chance to do it, or the students just did not record the procedure they did in the logbooks. Thirdly, some of the students had a chance to perform multiple needle sticks to find the central vein, whereas this possibility was denied to others. Some supervisors were very cautious about patient safety, because according to Mansfield et al., the complication rate of catheterization increased markedly when a physician attempted more than two needle passes [16]. The presence of different supervisor-student interactions may be a potential explanation for such variability, this is not available either.

In the future, new teaching program will incorporate teaching medical students to use ultrasound for CVC and we will evaluate the skill acquisition on that. Logbooks have been used as tools for assessment of procedural skills [17]. With proper design, both subjective and objective data can be included. We will ask supervisors to record students’ performance at the end of the procedure on the logbooks after communicating with students. This will make the data in the logbooks more complete and accurate. Meantime, students should make a better use of simulation and accelerate the learning curve for skill acquisition. Once their success rate improves, students’ self-reported satisfaction and confidence will improve in return.

## Conclusions

From the logbooks, they show that the success rate of CVC performed by our medical student is 67.3%, but their performance, satisfaction and confidence improved with cumulative clinical procedural experience.

## Abbreviations

CVC: Central venous catheterization; ASA physical status: American Society of Anesthesiologists physical status.

## Competing interests

The authors declare that they have no competing interests.

## Authors’ contributions

AC made substantial contributions to the study design and drafted the manuscript. CHL made substantial contributions to the study design and had helped analyze the data. KCC performed data acquisition and helped draft the manuscript. CCY drafted the manuscript and helped analyze the data. HMY performed data acquisition and helped draft the manuscript. SZF made substantial contributions to the concept of the study and revised the manuscript. WZS designed the study and revised the manuscript. All authors read and approved the final manuscript.

## Pre-publication history

The pre-publication history for this paper can be accessed here:

http://www.biomedcentral.com/1472-6920/14/168/prepub
